# Alarmin HMGB1 and Soluble RAGE as New Tools to Evaluate the Risk Stratification in Patients With the Antiphospholipid Syndrome

**DOI:** 10.3389/fimmu.2019.00460

**Published:** 2019-03-14

**Authors:** Valeria Manganelli, Simona Truglia, Antonella Capozzi, Cristiano Alessandri, Gloria Riitano, Francesca Romana Spinelli, Fulvia Ceccarelli, Silvia Mancuso, Tina Garofalo, Agostina Longo, Guido Valesini, Maurizio Sorice, Fabrizio Conti, Roberta Misasi

**Affiliations:** ^1^Dipartimento di Medicina Sperimentale, Sapienza Università di Roma, Rome, Italy; ^2^Reumatologia, Dipartimento di Medicina Interna e Specialità Mediche, Sapienza Università di Roma, Rome, Italy

**Keywords:** HMGB1, sRAGE, antiphospholipid syndrome, thrombosis, recurrent abortion

## Abstract

Antiphospholipid antibody syndrome (APS) is a systemic autoimmune disease characterized by arterial and/or venous thrombosis, pregnancy morbidity in the presence of circulating “anti-phospholipid antibodies” (aPL). One of the main target antigens of aPL is β_2_-glycoprotein I (β_2_-GPI). APS may occur as a primary syndrome or associated with Systemic Lupus Erythematosus (SLE). High Mobility Group Box 1 (HMGB1) is a nuclear non-histone protein which is secreted from different type of cells during activation and/or cell death and may act as a proinflammatory mediator through ligation to its receptors, including RAGE. There is accumulating evidence that HMGB1 contributes to the pathogenesis of inflammatory and autoimmune diseases, especially SLE. In a previous study we demonstrated increased serum levels of HMGB1 in both primary and secondary APS patients. In this work we analyzed: (i) *in vitro* whether anti-β_2_-GPI antibodies from APS patients may induce both a HMGB1 cellular relocation by activation of its putative receptor RAGE in platelets and monocytes and, (ii) *ex vivo*, serum levels of HMGB1/soluble RAGE (sRAGE) in APS patients and their possible correlation with clinical manifestations. Platelets and monocytes from healthy donors were incubated with affinity purified anti-β_2_-GPI antibodies. HMGB1 and RAGE expression were analyzed by Western Blot. Sera from 60 consecutive APS patients (primary or secondary), diagnosed according to the Sydney Classification Criteria, were enrolled. As a control, 30 matched healthy subjects were studied. Serum levels of HMGB1 and sRAGE were analyzed by Western Blot. *In vitro* results showed that anti-β_2_-GPI antibodies were able to induce RAGE activation and HMGB1 cellular relocation in both monocytes and platelets. HMGB1 and sRAGE serum levels were significantly increased in APS patients in comparison with healthy subjects (*p*<0.0001). Interestingly, APS patients with spontaneous recurrent abortion showed significantly higher levels of sRAGE; moreover, in APS patients a direct correlation between serum levels of HMGB1 and disease duration was detected. Our observations suggest that anti-β_2_-GPI antibodies may trigger RAGE activation and HMGB1 cellular relocation during APS. Monitoring these molecules serum levels may represent an useful tool to evaluate the pathogenesis and risk stratification of clinical manifestations in APS.

## Introduction

Antiphospholipid antibody syndrome (APS) is a systemic autoimmune disease characterized by arterial and/or venous thrombosis, pregnancy morbidity in the presence of “anti-phospholipid antibodies” (aPL) namely lupus anticoagulant (LA), anticardiolipin antibodies (aCL), or anti-β_2−_glycoprotein I antibodies (aβ_2_-GPI) (1). The incidence of the APS is around 5 new cases per 100,000 persons per year and the prevalence around 40–50 cases per 100,000 persons (2). APS may occur in patients having neither clinical nor laboratory evidence of another definible condition (primary APS, PAPS) or it may be associated with other diseases (secondary APS, SAPS), mainly Systemic Lupus Erythematosus (SLE) (3), but occasionally with other autoimmune conditions, infections (4), drugs (5), and malignancies (6).

There is accumulating evidence that High Mobility Group Box 1 (HMGB1), an endogenous danger signal released when immune cells are activated or cell death occurs (7), contributes to the pathogenesis of inflammatory and autoimmune diseases, especially SLE (8, 9). HMGB1 is a 30 kDa nuclear protein which was purified from nuclei for the first time in 1970 (10). In the nucleus it works as a chromatin structural protein, organizing DNA, stabilizing nucleosome formation and regulating transcription; it was also found in cytosol, mitochondria and cell plasma membrane, where it can be released to the extracellular milieu (11). In particular, HMGB1 may be secreted by immune cells, such as macrophages, monocytes, and dendritic cells, during activation and/or cell death (12, 13) and may act as a proinflammatory mediator through ligation to its receptors, Toll like receptor 2 or 4 (TLR2, TLR4) or its most interesting binding partner, Receptor for Advanced Glycation End products (RAGE) (14, 15). RAGE is a transmembrane receptor of the immunoglobulin superfamily that engages diverse ligands. Ligand-triggered RAGE-dependent cellular activation results in several effects, including activation of nuclear factor-kB (NF-kB), increased expression of cytokines and adhesion molecules, and induction of oxidative stress (16, 17). The C-truncated secretory isoform of RAGE, termed soluble RAGE (sRAGE), can be shedded by several cell types, including monocytes, and may neutralize the AGEs-mediated damage by acting as a decoy (18–20).

Level of HMGB1/sRAGE increases in plasma and serum of patients with inflammatory diseases associated with sepsis or thrombosis. In a previous study (21) we detected increased serum levels of HMGB1/sRAGE in subjects affected by pregnancy morbidity, as well as in a small group of patients with primary or secondary APS, suggesting that in these patients elevated levels of HMGB1/sRAGE may represent an alarm signal, indicating an increase of proinflammatory triggers.

Moreover, it has been demonstrated that platelet activation increases RAGE surface expression (22) and that HMGB1 may represent a master regulator of the prothrombotic cascade involved in the pathogenesis of deep venous thrombosis (23). Indeed, HMGB1 is involved in the prothrombotic cross-communication between platelets, monocytes and neutrophil granulocytes, thus promoting the thrombotic process.

In order to evaluate whether autoantibodies in APS may be able to activate HMGB1/sRAGE, in this work we have analyzed *in vitro* whether anti-β_2_-GPI antibodies from APS patients can induce a relocation of HMGB1 to the cytosol and activation of its putative receptor RAGE in platelets and monocytes from healthy donors. Furthermore, in order to evaluate any correlations between HMGB1/sRAGE and different APS clinical manifestations, we analyzed serum levels of these molecules in a larger cohort of patients with APS.

## Materials and Methods

### Isolation of Monocytes

Human peripheral blood mononuclear cells (PBMC) from buffy coat of healthy donors were isolated by Lymphoprep density-gradient centrifugation (Nycomed Pharma, Oslo, Norway). Cells were washed 3 times in phosphate buffered saline (PBS), pH 7.4, and were isolated by density-gradient separation (Lympholyte; Cedarlane, Hornby, Ontario, Canada). CD14+ monocytes were purified by incubation with anti-CD14–coated microbeads (Miltenyi Biotec, Bergisch Gladbach, Germany), followed by sorting with a magnetic device (MiniMacs Separation Unit; Miltenyi Biotec), according to the manufacturer's instructions.

The purity of the isolated monocytes was evaluated by staining with a fluorescein isothiocyanate (FITC)–conjugated anti-CD14 antibody against monocytes and analyzing stained cells by flow cytometry. The purity was higher than 95% CD14+. The viability of the monocytes was up to 99%, using the Trypan blue staining.

Before *in vitro* experiments, monocytes were cultured for 24 h, in RPMI 1640, containing 2 mM L-glutamine, 100 units/ml of penicillin, 100 mg/ml of streptomycin, and 250 pg/ml of Fungizone (Gibco, Grand Island, NY), in the absence of antioxidant agents, at 37°C in a humified atmosphere, containing 5% CO_2_.

### Platelets Preparation

Platelets were prepared from blood samples of healthy donors, with the addition of acid citrate dextrose (ACD) as anticoagulant. Platelet-rich plasma (PRP) was preliminary obtained from the whole blood by centrifugation at 150 × g for 15 min at 20°C.

PRP was centrifuged at 900 × g for 10 min at 20°C. Platelet-poor plasma (PPP) was removed and pellets were re-suspended in Tyrode's buffer, containing 10% (v:v) ACD. Then, after washing, pellets were re-suspended in Tyrode's buffer, containing Bovine Serum Albumine (BSA), 3 mg/ml.

Platelet counts were performed by a hemocytometer (Coulter, Beckman Coulter, Brea, California, USA); that leukocyte contamination was <1 leukocyte/10^7^ platelets. The purity of the isolated platelets was confirmed by staining with a fluorescein isothiocyanate (FITC)–conjugated anti-CD41 mAb (Beckman Coulter) and analyzing by flow cytometry (Coulter Epics, Beckman Coulter).

### Purification of Anti-β_2_-GPI Antibodies

Human anti**-**β_2_-GPI IgG were purified by affinity chromatography, as previously reported (24), from 3 patients who had been diagnosed as affected by APS according to the Sydney Classification Criteria (1), showing a high titer of anti-β_2_-GPI antibodies and, as a control, from 3 healthy donors. The antibodies displayed lupus anticoagulant (LA) activity, in all tests, the stimulatory effect of the 3 antibodies was virtually the same (data not shown).

### *In vitro* Incubation of Monocytes and Platelets With Anti-β_2_-GPI Antibodies

For *in vitro* studies, monocytes were cultured at 37°C in a humified atmosphere of 5% CO_2_ with serum-free RPMI 1640, containing 2 m*M* L-glutamine, 100 units/ml of penicillin, 100 mg/ml of streptomycin, and 250 pg/ml of Fungizone. Platelets were resuspended in Tyrode's buffer, containing BSA (3 mg/ml).

Monocytes (2 × 10^6^/ml) or platelets (300 × 10^6^/ml) were incubated at 37°C for 4 h with human affinity-purified anti-β_2_-GPI IgG (200 μg/ml), according to the method of Raschi et al. (25) with normal human serum IgG (200 μg/ml), or with LPS (100 ng/ml). All materials contained < 0.00025 ng of endotoxin/μg of protein, as determined by the *Limulus* amebocyte lysate test (Associates of Cape Cod, Falmouth, MA).

### Immunoblotting Analysis of HMGB1 Expression

In order to analyze HMGB1 relocation in cell compartments, monocytes, untreated or treated for 4 h at 37°C with normal human serum IgG, or with human affinity-purified anti-β_2_-GPI, or with LPS were subjected to subcellular fractionation according to Manganelli et al. (26). Equal amounts of nuclear or cytosolic extracts were separated by 12% SDS-PolyAcrylamide Gel Electrophoresis (SDS-PAGE). The proteins were electrophoretically transferred onto polyvinylidene fluoride (PVDF) transfer membranes (Amersham Biosciences, Piscataway, NJ, USA) and after blocking with Tris-buffered saline, that contains 25 mM Tris-HCl, 150 mM NaCl, pH 7.4, and 0.05% Tween-20 (TBS-T) with 3% BSA, were probed with rabbit anti-HMGB1 polyclonal Ab (1:1,000; Abcam, Cambridge UK). The secondary Ab was horseradish peroxidase conjugated anti-rabbit (1:1,0000; Sigma-Aldrich, Milan, Italy). After washing, proteins were detected using ECL reagents (Amersham Biosciences, Buckinghmashire, UK). As a control, anti-actin mAb antibodies (1:1,000 Sigma-Aldrich) and anti-LMNB1 mAb (1:1,000 Santa Cruz Biotechnology, Dallas, Texas, USA) were used. Lamin B1 (LMNB1) served as nuclear contamination marker and actin as cytoplasmic contamination marker. Densitometric analysis was performed using ImageJ Software (National Institutes of Health, Bethesda, MD, USA).

To analyze HMGB1 release from monocytes, supernatants of untreated and treated cells were analyzed by immunoblotting. The cells were stimulated for 24 h with human affinity-purified anti-β_2_-GPI. For Western blotting of HMGB1, supernatants were collected after 4 or 24 h of culture and concentrated by Centricon YM-10 (Millipore). The volume of the concentrated supernatants was adjusted to 70μl for equal loading. Samples and HMGB1 standard (Human recombinant HMGB1 SIGMA) were resolved in 12% SDS-PAGE under reducing conditions, as described before, using rabbit anti-HMGB1 polyclonal antibody (1:1,000; Abcam).

In parallel experiments platelets obtained from a healthy donor were incubated for 4 h at 37°C with normal human serum IgG, or with human affinity-purified anti-β_2_-GPI, or with LPS. Cells were lysed in Radioimmunoprecipitation assay (RIPA) buffer containing 1 mM Na_3_VO_4_, and 75 U of aprotinin, and analyzed by immunoblotting as described above using rabbit anti-HMGB1 polyclonal antibody (1:1,000; Abcam).

### Immunoprecipitation of RAGE

Monocytes and platelets, untreated or treated for 4 h at 37°C with normal human serum IgG, or with human affinity-purified anti-β_2_-GPI, or with LPS (27) were lysed in RIPA buffer 1 mM Na_3_VO_4_, and 75 U of aprotinin. To preclear non-specific binding, cell-free lysates were mixed with protein G-acrylic beads (Sigma-Aldrich) and stirred by a rotary shaker for 2 h at 4°C. The supernatants were centrifuged (500 × g for 1 min) and then immunoprecipitated with goat polyclonal anti-RAGE (1:1,000; Abcam) or with irrelevant IgG as a negative control plus protein G-acrylic beads. The immunoprecipitates were analyzed and checked by Western Blot.

### Immunoblotting Analysis of Phospho-RAGE Expression

Immunoprecipitated samples from monocytes and platelets untreated or treated as reported above, were subjected to immunoblotting analysis. After evaluation of the protein concentration by Bradford Dye Reagent assay (Bio-Rad, Segrate, Italy), the immunoprecipitates were analyzed by immunoblotting using mouse anti-Phospho-Ser mAb (1:1,000; Sigma-Aldrich), followed by a secondary Ab horseradish peroxidase conjugated anti-mouse (1:10,000; Sigma-Aldrich). Immunoprecipitation was checked by mouse anti-RAGE mAb (1:1,000; Millipore, Billerica, MA, USA). Densitometric analysis was performed using ImageJ Software (National Institutes of Health, Bethesda, MD, USA).

### Patients

Sixty consecutive adult patients classified as affected by APS according to the Sydney Classification Criteria (1), attending the Lupus Clinic, Sapienza University of Rome were consecutively enrolled.

Written informed consent was obtained from eligible patients during the screening period, at which time physical examination and medical history were evaluated.

In addition, 30 healthy subjects (normal blood donors, mean age ± *SD* 40.2 ± 8.8 F/M 25/5) were included as controls.

Subjects (both patients and controls) carrying any other disease, such as neurodegenerative, infectious or hepatic, were excluded, as well as pregnant or breast feeding women. In addition, subjects with surgical/anesthesia trauma in the last 3 months were also excluded. For control subjects, drugs interfering with hormonal, metabolic, or immunological function were also exclusion criteria.

Anti-CL and anti-β_2_-GPI antibodies were tested by enzyme-linked immunosorbent assay (ELISA) kits obtained from Inova Diagnostic Inc. (San Diego, CA, USA). Lupus Anticoagulant was studied by a dilute sensitized activated partial thromboplastin time (aPTT) and a dilute Russell's viper venom time (dRVVT), followed by confirm tests using reagents and instrumentation by Hemoliance Instrumentation Laboratory, Lexington, MA, USA.

This study was approved by the local ethic committees. Sera were collected at several times and stored at −20°C until use.

### Western Blot Analysis of HMGB1/sRAGE Levels in Sera

To avoid the possibility that serum/plasma components able to bind to HMGB1 may interfere with its detection, we tested HMGB1 by Western Blot, instead of ELISA (21, 28).

Briefly, sera (3 μl) from patients with APS or healthy donors were diluted with 72 μl RIPA buffer and heated at 95°C for 5 min in Sodium Dodecyl Sulfate (SDS)-loading buffer. For immunodetection, the proteins were separated by 12% SDS-PAGE and transferred onto PVDF transfer membranes. The membrane was blocked at room temperature for 1 h with TBS-T with 3% BSA. The membranes were incubated with primary Ab: anti-HMGB1 polyclonal Ab (1:1000; Abcam) or mouse anti-RAGE mAb (1:10,000; Millipore). The secondary Ab was horseradish peroxidase conjugated anti-rabbit (1:10,000; Sigma-Aldrich) or anti-mouse (1:5,000; Amersham Biosciences) IgG, which was incubated for 1 h at room temperature. After washing, proteins were detected using ECL reagents (Amersham Biosciences). Densitometric analysis was performed using ImageJ Image Software (National Institutes of Health).

### Statistical Analysis

All the statistical analysis were performed by GraphPad Prism software Inc. (San Diego, CA, USA). D'Agostino-Pearson omnibus normality test was used to assess the normal distribution of the data. Normally distributed variables were summarized using the mean ± standard deviation (SD) and non-normally distributed variables by the median and interquartile range (IQR). Frequencies were expressed by percentage. Paired *t*-test and Wilcoxon's matched pairs test were performed accordingly. *P* < 0.05 were considered statistically significant.

## Results

### Anti-β_2_-GPI Antibodies Induce HMGB1 Relocation and RAGE Phosphorylation in Monocytes

In order to evaluate whether autoantibodies in APS may be able to elicit the alarmin response, we preliminary analyzed *in vitro* whether anti-β_2_-GPI antibodies from APS patients may induce both a HMGB1 relocation to cytosol and an activation of its putative receptor RAGE in monocytes from healthy donors.

**Figure 1:**
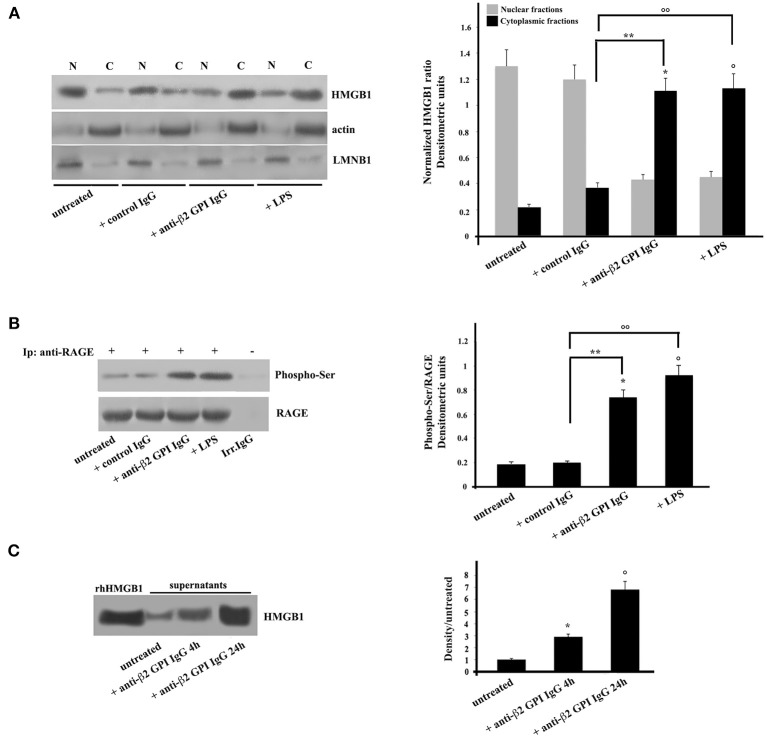
Anti-β_2_-GPI antibodies induce HMGB1 translocation and RAGE phosphorylation in human monocytes. **(A)** Monocytes from healthy donors, untreated or treated with control human IgG (200 μg/ml), human affinity-purified anti-β_2_-GPI IgG (200 μg/ml) or LPS (100 ng/ml), for 4 h at 37°C, were subjected to subcellular fractionation. Equal amounts of nuclear (N) or cytosolic (C) extracts were analyzed by immunoblotting using rabbit anti-HMGB1 polyclonal Ab. Lamin B1 (LMNB1) served as nuclear contamination marker and actin as cytoplasmic contamination marker. HMGB1 loading within each compartment was also normalized with Lamin B1 and actin, respectively. As a control, mouse anti-actin monoclonal antibodies and anti-LMNB1 mAb were used. Right panel, densitometric HMGB1 levels are shown. Results represent the mean ± SD from 3 independent experiments. ^*^p + anti-β_2−_GPI IgG < 0.001 vs. untreated, °*p* + LPS < 0.001 vs. untreated, ^**^p + anti-β_2−_GPI IgG < 0.001 vs. + control IgG, °°*p* +LPS < 0.001 vs. + control IgG. **(B)** Human monocytes from healthy donors, untreated or treated with control human serum IgG (200 μg/ml), with human affinity-purified anti-β_2_-GPI IgG (200 μg/ml) or LPS (100 ng/ml), for 4 h at 37°C, were immunoprecipitated with goat anti-RAGE polyclonal Ab. Immunoprecipitates were analyzed using mouse anti-Phospho-Ser monoclonal antibody. Immunoprecipitation was checked by mouse anti-RAGE mAb. Right panel, densitometric phospho-Ser /RAGE ratios are shown. Results represent the mean ± SD from 3 independent experiments. ^*^p + anti-β_2_-GPI IgG < 0.001 vs. untreated, °*p* + LPS < 0.001 vs. untreated, ^**^p + anti-β_2_-GPI IgG < 0.001 vs. + control IgG, °°*p* + LPS < 0.001 vs. + control IgG. **(C)** Western blot analysis of monocyte supernatants for HMGB1. The supernatants of untreated and cells treated for the indicated times with human affinity-purified anti-β_2_-GPI and HMGB1 standard (rhHMGB1) were run under reducing conditions on the gel (12% SDS-PAGE) and transferred to nitrocellulose. Filters were stained with rabbit anti-HMGB1 polyclonal Ab. Right panel, density/untreated is shown. Results represent the mean ± SD from 3 independent experiments. ^*^p + anti-β_2_-GPI IgG 4 h < 0.001 vs. untreated, °*p* + anti-β_2_-GPI IgG 24 h < 0.001 vs. untreated.

Purified monocytes, untreated or stimulated with anti-β_2_-GPI antibodies, or incubated with IgG from healthy donors or LPS as controls, were subjected to subcellular fractionation and equal amounts of nuclear or cytosolic extracts were analyzed by Western Blot. Our results revealed that the cytosolic fraction of monocytes incubated with anti-β_2_-GPI antibodies showed the relocation of HMGB1 from nuclei (Figure 1A). Virtually no relocation was observed following treatment with control IgG. As expected (29), treatment with LPS also induced relocation of HMGB1 from nuclei. Loading control was evaluated using anti-actin mAb as a cytosol protein marker and anti-LMNB1 as a nuclear protein marker. Densitometric analysis, reported on the right panel, clearly showed the effect of anti-β_2_-GPI antibodies on the HMGB1 relocation from nuclei to cytosol in monocytes.

Then, we analyzed the activation of the putative receptor of HMGB1, RAGE. Anti-β_2_-GPI antibodies were able to elicit in purified monocytes the activation of RAGE, which appeared in its phosphorylated form (30) (Figure 1B). Similar findings were found following LPS treatment, which has been reported to being involved in RAGE regulation (27). Virtually no RAGE phosphorylation was observed following treatment with control IgG. The identity of RAGE was confirmed using an anti-RAGE mAb (Figure 1B).

Since extracellular HMGB1 is known to be able to interact with RAGE, and in turn participate to the cell activation, i.e., inducing proinflammatory phenotype (12), we investigated whether HMGB1 was secreted from monocytes treated with anti-β_2_-GPI antibodies from APS patients. The level of HMGB1 was assessed by Western Blotting and by measuring the intensities of the immunoreactive bands at 25 kDa. As shown in Figure 1C, HMGB1 was significantly increased as early as after 4 h of stimulation. Densitometric analysis revealed higher concentration of HMGB1 in supernatant of cells stimulated with anti-β_2_-GPI antibodies from APS patients either for 4 or 24 h than in the supernatants of the untreated cells. This indicates that HMGB1 secretion by activated monocytes is a late event, as well documentated after LPS treatment (Figure 1C).

We further investigated weather mTORC1 activation is involved in aPL-induced secretion of HMGB1 and RAGE. We observed that anti-β_2_-GPI IgG from APS patients or starvation (HBSS) caused significant reduction in phospho-mTOR compared to untreated cells or to monocytes stimulated with control human serum IgG, *p* < 0.001 (no change was detected in the levels of total mTOR) (Supplementary Figure 1).

### Anti-β_2_-GPI Antibodies Induce HMGB1 Expression and RAGE Phosphorylation in Platelets

**Figure 2:**
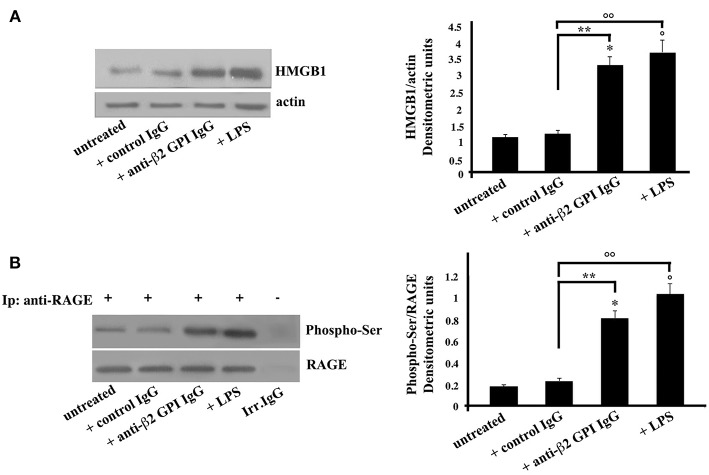
Anti-β_2_-GPI antibodies induce an increase of HMGB1 expression and RAGE activation in platelets. **(A)** Platelets from healthy donors, untreated or treated with control human serum IgG (200 μg/ml), human affinity-purified anti-β_2_-GPI IgG (200 μg/ml) or LPS (100 ng/ml) for 4 h at 37°C, were analyzed by Western Blotting using rabbit anti-HMGB1 polyclonal Ab. Expression of HMGB1was detected and representative images of three independent experiments were shown. Right panel: the ratio of HMGB1 immunopositivity to actin was calculated. The results are expressed as mean ± SD from 3 independent experiments. ^*^p + anti-β_2_-GPI IgG < 0.001 vs. untreated, °*p* + LPS < 0.001 vs. untreated, ^**^p + anti-β_2_-GPI IgG < 0.001 vs. + control IgG, °°*p* + LPS < 0.001 vs. + control IgG. **(B)** Human platelets, untreated, or treated as above were lysed in RIPA buffer. Cell lysates were prepared and immunoprecipitated with goat anti-RAGE polyclonal Ab. Immunoprecipitates were analyzed using mouse anti-Phospho-Ser mAb. Immunoprecipitation was checked by mouse anti-RAGE monoclonal Ab. Right panel, densitometric Phospho-Ser /RAGE ratios are shown. Results represent the mean ± SD from 3 independent experiments. ^*^p + anti-β_2_-GPI IgG < 0.001 vs. untreated, °*p* + LPS < 0.001 vs. untreated, ^**^p + anti-β_2_-GPI IgG < 0.001 vs. + control IgG, °°*p* + LPS < 0.001 vs. + control IgG.

In the same vein, platelets, as emblematic cells involved in the pathogenesis of thrombosis, were incubated with anti-β_2_-GPI antibodies. Again, similarly to monocytes, Western Blot analysis of HMGB1 on cell lysates obtained from anti-β_2_-GPI antibodies-treated platelets revealed that HMGB1 levels were significantly increased after 4 h of anti-β_2_-GPI treatment, as well as after LPS treatment (Figure 2A), as confirmed by densitometric analysis reported on the right panel. Loading control was evaluated using anti-actin mAb.

As showed in monocytes, also in platelets the anti-β_2_-GPI antibodies were able to elicit the activation of RAGE, indeed the Western Blot analysis of RAGE immunoprecipitated from platelets lysates showed the molecule in its serine phosphorylated form (Figure 2B). Similar findings were found following LPS treatment. Virtually no phosphorylation was observed following treatment with control IgG.

### Patients Characteristics and Clinical Manifestations

The 60 APS patients enrolled in this study were all Caucasian, 25 were primary APS and 35 APS associated with SLE. The clinical and demographic characteristics of the enrolled patients are reported in Table 1. No patient had liver enzyme elevations. The absolute number of circulating monocytes was in the normal range (0.1–1 × 10^−3^/μL) in each patient.

**Table 1:** Clinical characteristics of patients studied.

**Characteristics *n* (%)**	**APS patients (*n* = 60)**
F/M	51/9
Age (years)	
Mean ± SD	43.5 ± 13.3
Primary APS/APS associated with SLE	25/35
Disease duration (years) Median (IQR)	5 (11)
**AUTOANTIBODIES**	
aCL (IgG or IgM)	37 (61.7)
aβ2-GPI (IgG or IgM)	34 (56.7)
LA	35 (58.3)
Platelets Median (IQR)	243 (72)
**TREATMENTS**	
Oral anticoagulants	29 (48.3)
Antiaggregants	32 (53.3)
Glucocorticoids	17 (28.3)
Hydroxychloroquine	19 (31.7)
Azathioprine	5 (8.3)
Thalidomide	1 (1.7)
Sulphasalazyne	1 (1.7)
Pregnancy morbidity	18 (35)
Spontaneous abortions^*^	13 (25.5)
Normal Fetus Deaths^**^	4 (7.8)
Premature births^***^	3 (5.9)
Vascular thrombosis^#^	51 (85)
Arterial thrombosis	24 (40)
Venous thrombosis	37 (61.7)
Recurrent thrombosis	25 (41.7)
**NON-CRITERIA APS FEATURES**	
Livedo reticularis	18 (18.3)
Thrombocytopenia	13 (30)
Migraine	7 (21.7)
Seizures	8 (18.3)

* *3 or + losses<10 weeks of gestation*;

** *1 or + losses ≥10 weeks of gestation*;

*** *preterm birth <34 weeks due to eclampsia, pre-eclampsia, or placental insufficiency*.

# *Thrombosis (arterial, venous, or in small vessels) in any tissue, confirmed by imaging or histopathology (thrombosis without significant inflammation). aCL, anticardiolipin antibodies; aβ2GPI, anti-β2glycoprotein-I antibodies; LA, lupus anticoagulant; IQR, interquartile range*.

### Serum Levels of HMGB1 and sRAGE in APS Patients and Clinical Outcomes

Since in a previous work we detected increased serum levels of HMGB1 in subjects affected by pregnancy morbidity, as well as in a small group of patients with APS (21), in this investigation we wanted to extend the data evaluating serum levels of HMGB1/soluble RAGE (sRAGE) in patients with APS and their possible correlation with clinical manifestations.

**Figure 3:**
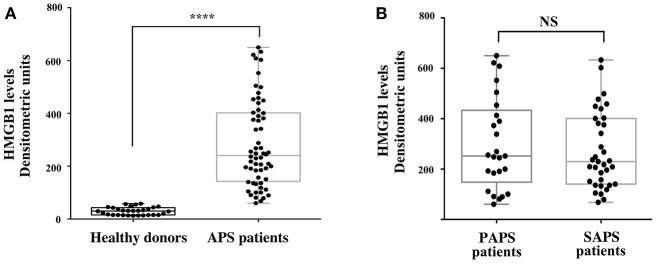
Increase of serum HMGB1 levels in APS patients. **(A)** HMGB1 level were detected by Western Blot in APS patients (*n* = 60) and healthy donors (*n* = 30) sera, using rabbit anti-HMGB1 polyclonal Ab. Densitometric values of HMGB1 levels calculated in healthy subjects and APS patients are represented and summarized by boxplot statistics. A statistically significant difference between expression of HMGB1 in APS patients vs. control subjects was found (^****^p < 0.0001). **(B)** Densitometric values of HMGB1 levels calculated in PAPS patients (*n* = 25) and SAPS patients (*n* = 35) are represented and summarized by boxplot statistics. NS, not significant.

The results showed that the APS patients, either PAPS or SAPS, showed significantly increased serum levels of HMGB1, as compared to healthy subjects (Figure 3A), as revealed by densitometric analysis (*p* < 0.0001). Furthermore, no significant differences of HMGB1 levels between primary and secondary APS were found (Figure 3B).

In APS patients with recurrent thrombosis, a tendency to have elevated HMGB1 serum levels has been highlighted, compared to APS patients without recurrent thrombosis, although this result does not reach statistical significance (Table 2). In APS patients a correlation between serum levels of HMGB1 and disease duration and age was detected (Table 3).

**Table 2:** Treatment influence on HMGB1 and sRAGE serum levels.

**Variable no vs. yes**	**HMGB1 Densitometric Unit median (IQR)**	***P-*value**
Antiaggregants	213.4 (151.5) vs. 331.93 (286.72)	0.042
Oral anticoagulants	251.89 (265) vs. 236.55 (236.89)	0.404
Glucocorticoids	278.52 (292.54) vs. 251.89 (265)	0.595
Hydroxychloroquine	206.57 (292.84) vs. 375.87 (221.79)	0.086
Azathioprine	278.52 (264.16) vs. 227.35 (254.48)	0.538
	**sRAGE Densitometric Unit median (IQR)**	***P*****-value**
Antiaggregants	1012.82 (488) vs. 1036.92 (289.92)	0.585
Oral anticoagulants	1034.73 (366) vs. 1022.78 (491.36)	0.678
Glucocorticoids	1095 (425.57) vs. 953.83 (293.74)	0.068
Hydroxychloroquine	952.71 (275.91) vs. 1133.87 (424.21)	0.085
Azathioprine	1039.12 (382.5) vs. 944.27 (488.89)	0.381

*HMGB1, High Mobility Group Box 1; sRAGE, serum Receptor for Advanced Glycation End products; IQR, Interquartile Range*.

**Table 3:** Univariate analysis with serum HMGB1 and sRAGE.

	**Spearman *r***	**95% CI**	***P*-value**
HMGB1(DU) vs. sRAGE (DU)	0.1390	−0.1268 to 0.3861	0.2897
HMGB1(DU) vs. Platelets (units/μl)	0.03958	−0.2884 to 0.3592	0.8109
HMGB1(DU) vs. Disease duration (years)	0.2801	0.01804 to 0.5061	0.0317
HMGB1(DU) vs. Age (years)	0.3608	0.1100 to 0.5684	0.0046
sRAGE (DU) vs. Platelets (units/μl)	−0.1419	−0.4457 to 0.1911	0.3888
sRAGE (DU) vs. Disease duration (years)	0.02738	−0.2377 to 0.2887	0.8369
sRAGE (DU) vs. Age (years)	−0.001654	−0.2627 to 0.2596	0.9900

*HMGB1, High Mobility Group Box 1; sRAGE, serum Receptor for Advanced Glycation End products; DU, Densitometric Units*.

**Figure 4:**
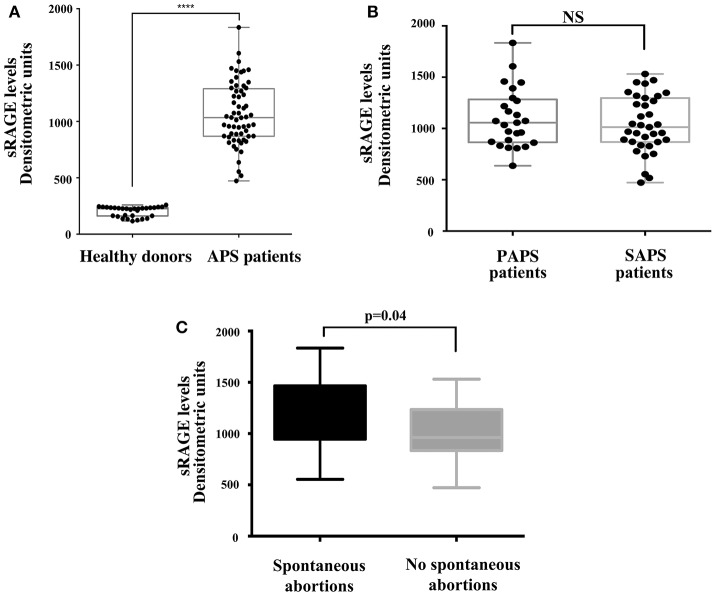
Increase of serum sRAGE levels in APS patients. **(A)** sRAGE levels were detected by Western Blot in APS patients (*n* = 60) and healthy donors (*n* = 30) sera, using mouse anti-RAGE mAb. Densitometric values of sRAGE levels calculated in healthy subjects and APS patients are represented and summarized by boxplot statistics. A statistically significant difference between expression of sRAGE in APS patients vs. control subjects was found (^****^p < 0.0001). **(B)** Densitometric values of sRAGE levels calculated in PAPS patients (*n* = 25) and SAPS patients (*n* = 35) are represented and summarized by boxplot statistics. NS, not significant. **(C)** Densitometric analysis of sRAGE levels, represented and summarized by boxplot statistics, revealed that patients with spontaneous abortions had significantly higher values than patients without spontaneous abortions (*p* = 0.04).

As for the analysis of sRAGE serum levels, it revealed that patients with APS showed increased serum levels of protein compared to healthy controls (Figure 4A). This difference was highly significant (*p* < 0.0001) while no significant differences in sRAGE levels were found between primary and secondary APS (Figure 4B). Interestingly, in APS patients with spontaneous recurrent abortions we observed increased levels of sRAGE compared to APS patients without recurrent abortions (*p* = 0.04) (Figure 4C).

No significant correlation between HMGB1 levels and sRAGE levels were found.

## Discussion

To analyze whether autoantibodies in APS may trigger the alarmin response, we preliminary analyzed *in vitro* whether anti-β_2_-GPI antibodies from APS patients may induce both a HMGB1 relocation and activation of its putative receptor RAGE. We observed that anti-β_2_-GPI can induce HMGB1 cellular relocation in both monocytes and platelets. Monocyte subpopulations in autoimmune diseases have been studied in the last few years. In particular, M1 subtype plays an important inflammatory role in SLE pathogenesis and reduced M2a and M2c subpopulations may contribute to the lack of anti-inflammatory activity, whereas M2b subtype may play a role in causing disease directly (31). No significant difference was observed in the proportion of monocyte subsets between patients with APS and patients with SLE (32), as well as in our case in the expression of HMGB1 among monocyte subsets (data not shown). Furthermore, the anti-β_2_-GPI Abs can induce the activation of RAGE, which appeared in its phosphorylated form. HMGB1 expression in monocytes may have several mechanistic implications. In particular, we observed that *in vitro* anti-β2-GPI-triggered HMGB1 release induces a decrease of phosphorylated mTOR. This finding is not surprising since it has already reported that HMGB1/RAGE knockdown is able to induce mTOR phosphorylation (33, 34), but may contribute to clarify the effect in APS patients of drugs able to induce mTOR blockade (35). Indeed, activation of the mTORC pathways plays a role in the vascular changes that are characteristic of APS nephropathy. Consequently, mTOR inhibitors such as sirolimus are currently used with a beneficial secondary effect on endothelial cells (35).

Regarding the molecular mechanisms by which anti-β_2_-GPI antibodies may trigger the HMGB1/RAGE pathway in human monocytes, we can hypothesize that the antibodies may react with their target antigen strictly in association with TLR4 (24). Because of the molecular mimicry among β_2_-GPI and bacterial antigens or microbial products, β_2_-GPI might directly interact with a TLR (24) or, alternatively, anti-β_2_-GPI may crosslink the molecule and TLR4 (36), thus inducing a proinflammatory and procoagulant phenotype, as ourselves already reported. As reported in results, HMGB1 was significantly increased in supernatant of cells stimulated with anti-β_2_-GPI antibodies from APS patients either than in the supernatants of the untreated cells. It is well known that HMGB1 release by macrophages happens through the regulation of HMGB1 acetylation. In particular, in monocytes and macrophages HMGB1 can be extensively acetylated in such a way that the protein can be transferred from the nucleus to the cytoplasm and, in this form, be actively released by the secretory lysosomes (37). Thus, we suggest that most of HMGB1 in supernatant is acetylated. Alternatively, HMGB1 may be released along with NETosis, together with many other danger-associated molecular patterns in response to infections. Indeed, alarmins, including HMGB1, are detected as the initiators of NETosis (38).

Furthermore, HMGB1 was significantly increased in platelets„ with consequent RAGE activation, following anti-β_2_-GPI antibody triggering. These data are not surprising since it has been already reported (39) that progenitor cells provided PLT with HMGB1, mRNA as well as protein. This gives PLTs, once stimulated, the ability to increase the expression of HMGB1 from mRNA and eventually release it. Moreover, in patients HMGB1 expression on platelets might also be achieved by fusion of HMGB1 containing microparticles with the plasma membrane of active platelets (39).

A series of studies have provided a close link between HMGB1 and platelet activation. In 2000, Rouhiainen et al. (40) detected the expression of HMGB1 in human platelets, using the Western blot and reverse transcription-polymerase chain reaction. Today, there are many data indicating that inflammation may induce thrombosis through the activation of platelets (41) and studies of recent years have shed light on the functions of platelets activated by HMGB1. Over the last decade, increasing evidence has suggested that HMGB1 could recognize its receptors on platelets, contributing to platelet aggregation and secretion (42, 43). In fact, HMGB1 has been shown to be released outside stressed or activated cells, including platelets (7, 44). Once in the extracellular space, HMGB1 can exert its biological functions by interacting with its receptors. Stark et al. (23) identified a high expression of HMGB1 as an important regulator of the prothrombotic cascade involving myeloid leukocytes and platelets, that favor the formation of occlusive deep venous thrombosis in a mouse model of venous thrombosis induced by reduction of flow in the inferior vena cava. These *in vivo* data are in line with what we report in this paper, in fact we suggest the possibility in the APS of a role of anti-β_2_-GPI antibodies in the activation of the thrombotic cascade via the HMGB1 platelet pathway. Furthermore, again, in an *in vivo* model, Vogel et al. (45) reported that mice lacking HMGB1 in platelets showed increased bleeding times and reduced thrombus, platelet aggregation, inflammation and organ damage during experimental trauma/hemorrhagic shock. On the other hand, they showed that platelets were the main source of HMGB1 in the thrombi and that in traumatized patients, the expression of HMGB1 on the surface of the circulating platelets was markedly upregulated. Our data greatly reinforce the hypothesis considering platelet-derived HMGB1 as a potential target for the prevention of thrombosis.

In addition, following previous studies, by Shao et al. (46) in which is reported that preeclamptic sera and aPL both induced an increase in the cytoplasmic levels of the alarmin HMGB1, and by ourselves (21) in which we observed a significant increase in HMGB1 in sera from patients with APS, in the present work we confirmed and expanded these results in a larger cohort of patients with APS, demonstrating a significant correlation with the duration of the disease, without a significant difference between primary and secondary APS, which showed HMGB1 levels comparable with those detected in SLE patients (without APS) (47, 48).

Furthermore, in sera from APS patients with recurrent abortions we showed a significant increase in sRAGE respect to patients without. These findings are not surprising, since during pregnancy proinflammatory stimuli have been associated with higher risk of adverse pregnancy outcomes (49). In particular, HMGB1 may convey danger signals by triggering inflammatory patterns with extracellular signal–regulated kinases (ERKs), p38 and NF-kB activation via several cell surface receptors, including RAGE (22). Ota et al. showed that elevated levels of serum sRAGE are associated with recurrent pregnancy losses (RPL) and speculated that RAGE might contribute to RPL by reducing uterine blood flow and subsequently causing ischemia in the fetus via inflammatory and thrombotic reactions (50). Interestingly, our results also showed that, at a distance of the acute clinical manifestation, a significant increase in the serum level of the soluble form of RAGE persisted. This situation indicates the involvement of this molecular system in the pathogenesis of thrombotic episodes related to APS. Furthermore, the significant increase in serum sRAGE levels was observed in patients with spontaneous recurrent abortions, which represent one of the main manifestations of the syndrome.

In conclusion, although it is already widely known that HMGB1 can induce and improve innate immunity, playing a role in the inflammatory phenomena of autoimmune diseases, our findings suggest that, in subjects with APS, not only elevated levels of HMGB1 but also of sRAGE may represent an alarm signal, indicating an increase of proinflammatory triggers. Although this finding cannot be considered highly specific (21), HMGB1/sRAGE may play a role in monitoring of recurrent abortion risk. In addition, HMGB1 may be useful in monitoring patients during particular treatments, such as antiaggregants, in which we found significantly higher levels of HMGB1. Further studies are needed to demonstrate that monitoring HMGB1/sRAGE, together with other prognostic parameters, may be a useful tool to evaluate the risk stratification of clinical manifestation(s) during APS. Finally, our *in vitro* data suggest that monitoring of these molecules can be useful to evaluate some pathogenic steps of the clinical manifestations of APS.

## Ethics Statement

Ethics committee, Sapienza Università di Roma - Policlinico Umberto I. All the patients signed an informed consent prior to enter in the study.

## Author Contributions

VM, ST, MS, and RM conceived and designed the study. VM, AC, and GR performed the experiment. VM, ST, SM, FS, and FuC analyzed the data. MS and RM wrote the original manuscript. FaC, CA, GV, AL, and TG read and approved the final version of the manuscript.

### Conflict of Interest Statement

The authors declare that the research was conducted in the absence of any commercial or financial relationships that could be construed as a potential conflict of interest.

## Supplementary Material

Supplementary Figure 1 Anti-β_2_-GPI IgG induced decrease of mTOR phosphorylation in monocytes. Cells, untreated or treated with control human serum IgG (200 μg/ml), with human affinity-purified anti-β_2_-GPI IgG (200 μg/ml) or starved in total starvation medium (HBSS solution containing 1% rich medium), for 4 h at 37°C were lysed in RIPA buffer, containing protease and phosphatase inhibitors. Western blot analysis was done to detect the levels of phosphorylated (p-mTOR) and non-phosphorylated mTOR. The membrane was incubated with the following primary antibodies: rabbit polyclonal anti-p-mTOR (Ser2448; 1:1,000 Cell Signaling Technology, Inc., Boston, MA, USA) and anti-actin mAb antibodies (1:1,000 Sigma-Aldrich). The nitrocellulose membrane was stripped and re-probed for total mTOR levels using rabbit polyclonal anti-mTOR (1:1,000; Cell Signaling Technology, Inc.). Loading control was performed by evaluating actin expression in the same filter. Right panel, ratios of p-mTOR to mTOR were normalized to untreated, set at 1.0. Data are presented as the mean ± SD of three independent experiments. ^*^*p* + anti-β_2_-GPI IgG < 0.001 vs. untreated, °*p* + HBSS < 0.001 vs. untreated, ^**^*p* + anti-β_2_-GPI IgG < 0.001 vs. + control IgG, °°*p* + HBSS < 0.001 vs. + control IgG.
