# Supplementary figures and images for: Alarmin HMGB1 and Soluble RAGE as New Tools to Evaluate the Risk Stratification in Patients With the Antiphospholipid Syndrome
Source: Front Immunol. 2019 Mar 14;10:460. doi: 10.3389/fimmu.2019.00460 (PMC6426766; doi:10.3389/fimmu.2019.00460)

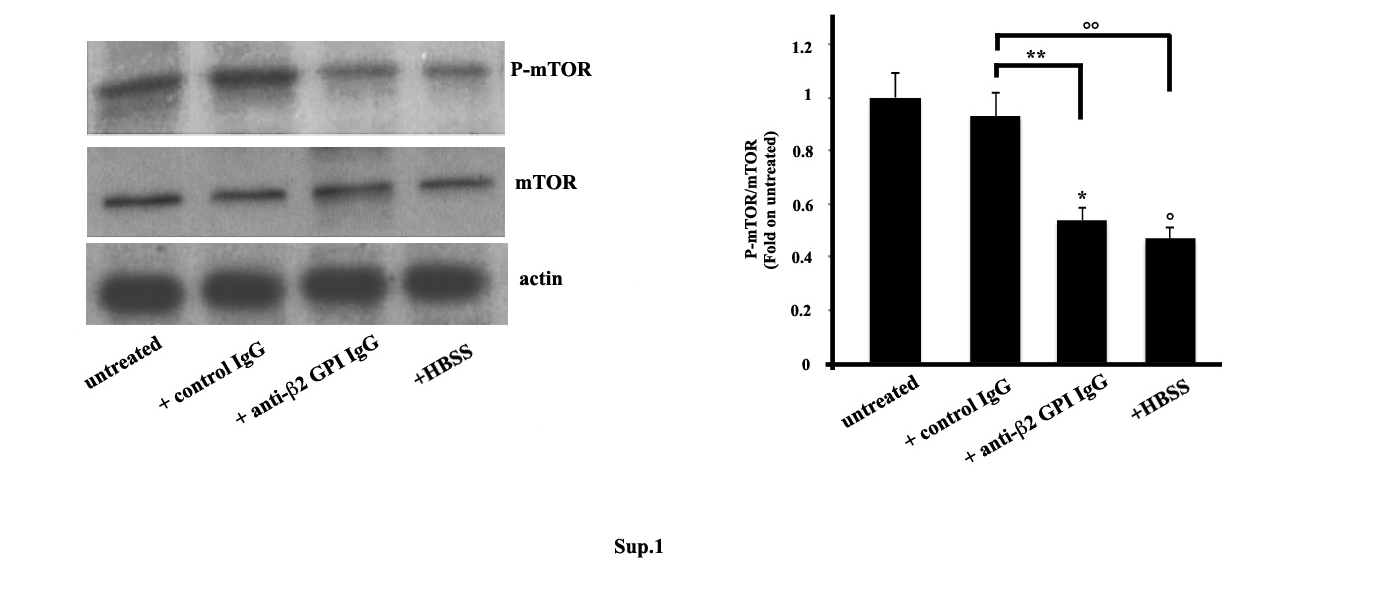

Supplement: Supplementary Figure 1 — Anti-β2-GPI IgG induced decrease of mTOR phosphorylation in monocytes. Cells, untreated or treated with control human serum IgG (200 μg/ml), with human affinity-purified anti-β2-GPI IgG (200 μg/ml) or starved in total starvation medium (HBSS solution containing 1% rich medium), for 4 h at 37°C were lysed in RIPA buffer, containing protease and phosphatase inhibitors. Western blot analysis was done to detect the levels of phosphorylated (p-mTOR) and non-phosphorylated mTOR. The membrane was incubated with the following primary antibodies: rabbit polyclonal anti-p-mTOR (Ser2448; 1:1,000 Cell Signaling Technology, Inc., Boston, MA, USA) and anti-actin mAb antibodies (1:1,000 Sigma-Aldrich). The nitrocellulose membrane was stripped and re-probed for total mTOR levels using rabbit polyclonal anti-mTOR (1:1,000; Cell Signaling Technology, Inc.). Loading control was performed by evaluating actin expression in the same filter. Right panel, ratios of p-mTOR to mTOR were normalized to untreated, set at 1.0. Data are presented as the mean ± SD of three independent experiments. *p + anti-β2-GPI IgG < 0.001 vs. untreated, °p + HBSS < 0.001 vs. untreated, **p + anti-β2-GPI IgG < 0.001 vs. + control IgG, °°p + HBSS < 0.001 vs. + control IgG. [file Image_1.TIFF]
